# Extracorporeal membrane oxygenation for postpneumonectomy ARDS

**DOI:** 10.1590/S1806-37132014000200018

**Published:** 2014

**Authors:** Maurício Guidi Saueressig, Patrícia Schwarz, Rosane Schlatter, Alexandre Heitor Moreschi, Orlando Carlos Belmonte Wender, Amarilio Vieira de Macedo-Neto

**Affiliations:** Department of Surgery, Porto Alegre Hospital de Clínicas, Federal University of Rio Grande do Sul School of Medicine, Porto Alegre, Brazil; Department of Intensive Care, Porto Alegre Hospital de Clínicas, Federal University of Rio Grande do Sul School of Medicine, Porto Alegre, Brazil; Graduate Program in Cardiology and Cardiovascular Sciences, Federal University of Rio Grande do Sul School of Medicine, Porto Alegre, Brazil; Department of Thoracic Surgery, Porto Alegre Hospital de Clínicas, Federal University of Rio Grande do Sul School of Medicine, Porto Alegre, Brazil; Department of Surgery, Porto Alegre Hospital de Clínicas, Federal University of Rio Grande do Sul School of Medicine, Porto Alegre, Brazil; Department of Surgery, Porto Alegre Hospital de Clínicas, Federal University of Rio Grande do Sul School of Medicine, Porto Alegre, Brazil

## To the Editor:

**Figure 1:**
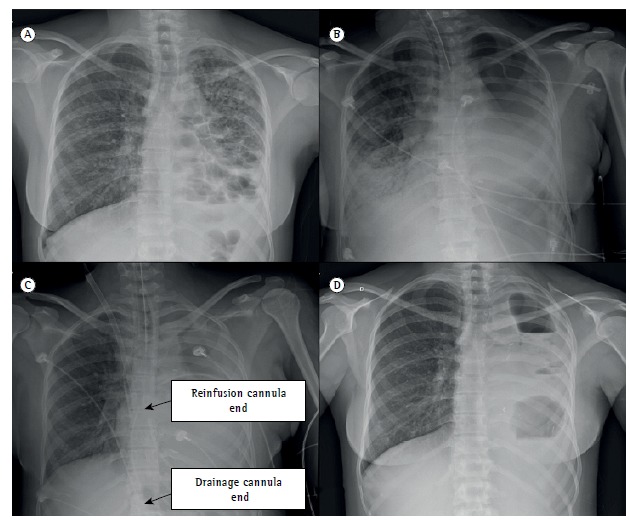
Chest X-rays showing the progression of the patient. In A, chest X-ray taken before pneumonectomy, showing extensive bronchiectasis, reduced lung volume, and left pleural thickening. In B, chest X-ray taken on postoperative day 3, showing extensive areas of consolidation on the right and the postpneumonectomy pleural space on the left. In C, chest X-ray taken on the day of weaning from extracorporeal membrane oxygenation (i.e., on postoperative day 8), showing resolution of the right-sided consolidation. Note the venous cannulae and their ends in the right atrium (for reinfusion) and in the intrahepatic portion of the inferior vena cava (for drainage). In D, chest X-ray taken three months after hospital discharge, showing nearly complete closure of the pneumonectomy cavity.

Although ARDS is an uncommon complication of pneumonectomy, the associated mortality is high (ranging from 50% to 100%).^(^ ^1^ ^)^ Here, we report the case of a patient with postpneumonectomy ARDS that was satisfactorily managed by extracorporeal membrane oxygenation (ECMO). A 31-year-old White female patient diagnosed with cystic fibrosis 10 years prior presented with recurrent pneumonia secondary to bronchiectasis, predominantly in the left lung (Figure 1A). In the last two years, despite continuous use of antibiotics (500 mg of azithromycin p.o. three times a week), the patient had had seven respiratory infections, as well as purulent sputum between episodes. Therefore, a decision was made to perform a left pneumonectomy. The postoperative course was satisfactory. However, on postoperative day 2, the patient showed dyspnea, cough with purulent sputum, tachypnea, left-sided chest pain, inspiratory rales in the lower lung fields, and hypoxemia (SaO_2_ < 70%). Initial management with noninvasive ventilation was ineffective, the patient being therefore placed on mechanical ventilation on postoperative day 3 (Figure 1B). After 17 h of mechanical ventilation, she still had ARDS, hypoxemia, and a pH < 7.2, despite alveolar recruitment maneuvers, attempts to reduce tidal volume, oxygen insufflation into the trachea, and neuromuscular blockade (Table 1). Therefore, a decision was made to place her on venovenous ECMO (a Revolution^TM^ centrifugal pump and an EOS ECMO adult membrane oxygenator; Sorin, Milan, Italy). Percutaneous cannulation of the femoral and jugular veins was performed by the Seldinger technique, a 19-F arterial cannula being inserted into the right internal jugular vein for reinfusion and a 29-F venous cannula being inserted into the right femoral vein for drainage (Maquet, Rastatt, Germany). Venipuncture and cannulation of the jugular and femoral veins were performed under ultrasound guidance at the bedside. Continuous i.v. infusion of unfractionated heparin was used in order to achieve an activated clotting time of 160-200 s. Initially, ECMO blood flow was 60 mL . kg^-1^ . min^-1^, being subsequently adjusted to maintain a PaO_2_ > 50 mmHg, whereas gas flow (sweep gas) was titrated to maintain a pH = 7.3. The temperature of the patient remained at 35.5-36.5°C. Lung rest was achieved by pressure-controlled ventilation at protective ventilator settings (i.e., a plateau pressure = 25 cmH_2_O, a positive end-expiratory pressure of 5-15 cmH_2_O, and an FiO_2_ = 0.4). The patient showed progressive radiological improvement (Figure 1C), as well as progressive improvement in arterial blood gas parameters and lung compliance, meeting the criteria for weaning on ECMO day 5 (Table 1). Three hours later, she was successfully extubated. The patient was discharged on postadmission day 21. There were no hemorrhagic or thromboembolic complications of ECMO. The total cost of ECMO, in Brazilian reals (R$), was 33,470.16, R$ 26,315.00 having been spent on the ECMO circuit plus medical supplies (including cannulae), R$ 5,594.93 having been spent on the ICU stay, and R$ 1,560.23 having been spent on diagnostic tests. However, the amount paid by the *Sistema Único de Saúde* (SUS, Brazilian Unified Health Care System) via the Authorized Hospital Admissions system was R$ 5,917.88.

**Table 1:** Clinical status before initiation of and on the day of weaning from extracorporeal membrane oxygenation.

Arterial blood gas results	Before ECMO	On the day of weaning from ECMO
pH	7	7.5
PaO_2_, mmHg	108	136
PaCO_2_, mmHg	115	57
PaO_2_/FiO_2_	107	388
Mechanical ventilation		
PEEP, cmH_2_O	8	6
FiO_2_	1.00	0.35
RR, breaths/min	28	15
Tidal volume/ideal weight, mL/kg	4.4	5.8
Plateau pressure, cmH_2_O	35	22
Peak inspiratory pressure, cmH_2_O	45	26
Static lung compliance, mL/cmH_2_O	9	20
Hemodynamics		
Norepinephrine, μg . kg^-1^ . min^-1^	0.16	0.00
Mean arterial pressure, mmHg	66	90
HR, bpm	90	80
Serum test results		
Lactate, mmol/L	0.4	0.9
Base excess, mmol/L	-3.5	17.0
C-reactive protein, mg/L	231	17
Hemoglobin, g/dL	10	9
Sedation/neuromuscular blockade/scores		
Midazolam, mg . kg^-1^ . h^-1^	0.2	0
Fentanyl, μg . kg^-1^ . h^-1^	4	2
Atracurium, mg . kg^-1^ . h^-1^	0.6	0.0
SOFA score	14	7
Murray score	3.0	1.2

ECMO : extracorporeal membrane oxygenation

PEEP : positive end-expiratory pressure

SOFA : Sequential Organ Failure Assessment

The incidence of ARDS after left pneumonectomy is approximately 4%.^(^ ^2^ ^)^ Possible triggers include reduced lymphatic drainage and single-lung ventilation with hyperoxia.^(^ ^3^ ^)^ Supportive care consists of mechanical ventilation; however, in cases of refractory hypoxemia, rescue therapies include prone positioning^(^ ^4^ ^)^ and ECMO.^(^ ^5^ ^)^

An invasive method, ECMO corrects severe hypoxemia and hypercapnia (pH = 7.2) and reduces FiO_2_ (< 0.5) and plateau pressure to safer levels, allowing the lung to rest in cases of ARDS.^(^ ^6^ ^,^ ^7^ ^)^ Despite a PaO_2_/FiO_2_ ratio > 100 mmHg, early ECMO was recommended because of the presence of an FiO_2_ > 0.8, a plateau pressure > 30 cmH_2_O, a pH < 7.2, and a PaCO_2_ > 100 mmHg in our patient. In addition, her Sequential Organ Failure Assessment score was 14, indicating the absence of multiorgan involvement and showing that the ECMO team at the Porto Alegre *Hospital de Clínicas*, located in the city of Porto Alegre, Brazil, abides by the policy that ARDS patients who are not at risk of imminent death should be recognized as candidates for ECMO. This approach has been advocated by other ECMO teams in Brazil.^(^ ^8^ ^,^ ^9^ ^)^

A novel technique, ECMO is currently not covered by the SUS; the reimbursement that our hospital received from the SUS covered less than 20% of the actual costs. Therefore, there is a need for an economic evaluation of ECMO in Brazil in order to inform the decision of whether ECMO should be included in the range of procedures covered by the SUS.

On the basis of the case reported here, we recommend early rescue therapy with venovenous ECMO for patients with postpneumonectomy ARDS accompanied by hypoxemia and respiratory acidosis refractory to mechanical ventilation, provided that the medical team has sufficient experience with the procedure, which is complex and costly.
