# Supplementary material for: Extracorporeal membrane oxygenation for postpneumonectomy ARDS
Source: J Bras Pneumol. 2014 Mar-Apr;40(2):203–6. doi: 10.1590/S1806-37132014000200018 (PMC4083648; doi:10.1590/S1806-37132014000200018)
Supplement: Translation

# Translation: Oxigenação extracorpórea por membrana no tratamento da SARA pós-pneumonectomia

**Authors:** Maurício Guidi Saueressig, Patrícia Schwarz, Rosane Schlatter, Alexandre Heitor Moreschi, Orlando Carlos Belmonte Wender, Amarilio Vieira de Macedo-Neto

**Affiliations:** Departamento de Cirurgia, Hospital de Clínicas de Porto Alegre, Faculdade de Medicina, Universidade Federal do Rio Grande do Sul, Porto Alegre (RS) Brasil; Serviço de Medicina Intensiva, Hospital de Clínicas de Porto Alegre, Faculdade de Medicina, Universidade Federal do Rio Grande do Sul, Porto Alegre (RS) Brasil; Programa de Pós-Graduação em Cardiologia e Ciências Cardiovasculares, Faculdade de Medicina, Universidade Federal do Rio Grande do Sul, Porto Alegre (RS) Brasil; Serviço de Cirurgia Torácica, Hospital de Clínicas de Porto Alegre, Faculdade de Medicina, Universidade Federal do Rio Grande do Sul, Porto Alegre (RS) Brasil; Departamento de Cirurgia, Hospital de Clínicas de Porto Alegre, Faculdade de Medicina, Universidade Federal do Rio Grande do Sul, Porto Alegre (RS) Brasil; Departamento de Cirurgia, Hospital de Clínicas de Porto Alegre, Faculdade de Medicina, Universidade Federal do Rio Grande do Sul, Porto Alegre (RS) Brasil

## Ao Editor:

A SARA pós-pneumonectomia é uma complicação infrequente, porém com alta mortalidade (de 50% até 100%).^(^ ^1^ ^)^ Gostaríamos de relatar um caso manejado satisfatoriamente com *extracorporeal membrane oxygenation* (ECMO, oxigenação extracorpórea por membrana). Uma mulher branca de 31 anos, com diagnóstico de fibrose cística havia 10 anos, apresentava pneumonias repetidas e secundárias a bronquiectasias que predominavam no pulmão esquerdo (Figura 1A). Nos últimos dois anos, apesar do uso ininterrupto de antibiótico (azitromicina, 500 mg v.o., três vezes por semana), apresentou sete infecções respiratórias, além de escarro purulento entre essas exacerbações. Dessa maneira, decidiu-se pela pneumonectomia esquerda. A evolução foi satisfatória até o 2º dia de pós-operatório (PO), quando ela iniciou com dispneia, tosse com escarro purulento, taquipneia, dor torácica em hemitórax à esquerda, estertores teleinspiratórios em campo pulmonar inferior e hipoxemia (SaO_2_ < 70%). O manejo inicial com ventilação não invasiva foi ineficaz, o que levou à instalação de ventilação mecânica (VM) no 3º PO (Figura 1B). Após 17 h de VM, a paciente mantinha-se com SARA, com hipoxemia e pH < 7,2, apesar das manobras de recrutamento alveolar, tentativas de redução do volume corrente, insuflação de oxigênio pela traqueia e bloqueio neuromuscular (Tabela 1). Assim, foi indicada a colocação da ECMO venovenosa (bomba centrífuga Revolution^TM^ e oxigenador adulto EOS ECMO; Sorin, Milão, Itália) com canulação femorojugular percutânea pela técnica de Seldinger - cânula arterial 19 F na veia jugular interna direita como via de reinfusão; cânula venosa 29 F na veia femoral direita como via de drenagem (Maquet, Rastatt, Alemanha). As punções venosas e o posicionamento intravascular das cânulas foram realizados sob orientação ecográfica à beira do leito. Definimos o tempo de coagulação ativada entre 160 e 200 segundos através de heparina não fracionada em infusão contínua i.v. O fluxo de sangue da ECMO, que inicialmente era de 60 mL . kg^-1^ . min^-1^, foi posteriormente ajustado para manter a PaO_2_ > 50 mmHg, enquanto o fluxo de gás (*sweep gas*) foi titulado a fim de sustentar um pH = 7,3. A temperatura da paciente permaneceu de 35,5-36,5°C. No mesmo momento, iniciamos o repouso pulmonar a partir da VM sob pressão controlada com parâmetros protetores (pressão de platô = 25 cmH_2_O, pressão expiratória final positiva de 5-15 cmH_2_O e FiO_2_ = 0,4). A paciente apresentou melhora radiológica (Figura 1C), gasométrica e da complacência pulmonar progressivamente e, no 5º dia de suporte extracorpóreo, preencheu os critérios de desmame da ECMO (Tabela 1). Três horas após, foi extubada com sucesso. A alta hospitalar ocorreu 21 dias após a internação. Não houve complicações hemorrágicas ou tromboembólicas associadas à ECMO. Em relação aos custos preliminares da ECMO, foram gastos R$ 26.315,00 com o circuito de oxigenação extracorpóreo, cânulas e materiais médico-hospitalares; R$ 5.594,93 com diárias do CTI; e R$ 1.560,23 com exames diagnósticos. O total gasto foi de R$ 33.470,16. No entanto, verificou-se que o valor faturado pela autorização de internação hospitalar do Sistema Único de Saúde (SUS) consistiu em apenas R$ 5.917,88.

**Figura 1:** Exames radiológicos que ilustram a evolução da paciente. Em A, radiografia de tórax no pré-operatório de pneumonectomia, com extensas bronquiectasias, redução de volume pulmonar e espessamento pleural à esquerda. Em B, radiografia no 3º dia de pós-operatório, com extensas consolidações à direita e cavidade pleural de pneumonectomia à esquerda. Em C, radiografia no dia do desmame da oxigenação extracorpórea por membrana, no 8º dia de pós-operatório, demonstrando regressão das consolidações à direita. Observam-se as cânulas venosas com as respectivas extremidades no átrio direito (linha de reinfusão) e na veia cava inferior na porção intra-hepática (linha de drenagem). Em D, radiografia retirada três meses após a alta hospitalar, demonstrando a cavidade de pneumonectomia à esquerda quase totalmente obliterada.

**Tabela 1:** Condição clínica antes da implantação e no dia de retirada da oxigenação extracorpórea por membrana.

Gasometria arterial	Pré-ECMO	Desmame
pH	7	7,5
PaO_2_, mmHg	108	136
PaCO_2_, mmHg	115	57
PaO_2_/FiO_2_	107	388
Ventilação mecânica		
PEEP, cmH_2_O	8	6
FiO_2_	1,00	0,35
FR, ciclos/min	28	15
Volume corrente/peso ideal, mL/kg	4,4	5,8
Pressão de platô, cmH_2_O	35	22
Pressão de pico inspiratório, cmH_2_O	45	26
Complacência pulmonar estática, mL/cmH_2_O	9	20
Hemodinâmica		
Noradrenalina, μg . kg^-1^ . min^-1^	0,16	0,00
Pressão arterial média, mmHg	66	90
FC, bpm	90	80
Exames séricos		
Lactato, mmol/L	0,4	0,9
Excesso de base, mmol/L	-3,5	17,0
Proteína C reativa, mg/L	231	17
Hemoglobina, g/dL	10	9
Sedação/bloqueio neuromuscular/escores		
Midazolam, mg . kg^-1^ . h^-1^	0,2	0
Fentanil, μg . kg^-1^ . h^-1^	4	2
Atracúrio, mg . kg^-1^ . h^-1^	0,6	0,0
SOFA	14	7
Escore de Murray	3,0	1,2

ECMO ; *extracorporeal membrane oxygenation* (oxigenação extracorpórea por membrana)

PEEP : *positive end-expiratory pressure* (pressão expiratória final positiva)

SOFA : *Sequential Organ Failure Assessment*

A incidência de SARA após a pneumonectomia esquerda é de aproximadamente 4%.^(^ ^2^ ^)^ Podemos citar como possíveis fatores desencadeantes a redução da drenagem linfática e a ventilação monopulmonar com hiperóxia, entre outros.^(^ ^3^ ^)^ Seu tratamento de suporte é centrado na VM; entretanto, nos casos de hipoxemia refratária, métodos como a pronação^(^ ^4^ ^)^ e a ECMO^(^ ^5^ ^)^ surgem como alternativas de resgate.

A ECMO é um método invasivo que corrige a hipoxemia grave e a hipercapnia com pH = 7,2, enquanto proporciona reduções da FiO_2_ (< 0,5) e da pressão de platô para níveis mais seguros, permitindo um maior repouso pulmonar em casos de SARA.^(^ ^6^ ^,^ ^7^ ^)^ Em nossa paciente, a presença de parâmetros altos de VM (FiO_2_ > 0,8 e pressão de platô > 30 cmH_2_O), associada a um pH < 7,2 com PaCO_2_ > 100 mmHg, levaram à indicação precoce da ECMO, mesmo que a relação PaO_2_/FiO_2_ estivesse acima de 100 mmHg. Além disso, o valor calculado de 14 para o índice *Sequential Organ Failure Assessment* reflete a ausência de comprometimento multiorgânico de nossa paciente, demonstrando a política da equipe de ECMO do Hospital de Clínicas, localizado na cidade de Porto Alegre (RS), que busca o reconhecimento precoce de pacientes com SARA candidatos à ECMO que não se encontrem ainda em eminente risco de óbito. Essa conduta já foi defendida por outros grupos brasileiros de ECMO.^(^ ^8^ ^,^ ^9^ ^)^

Em relação aos custos, a ECMO é uma nova tecnologia ainda não contemplada pelo SUS que, por sua vez, repassou ao hospital um valor que ressarciu menos de 20% daquele gasto com o respectivo tratamento. Dessa maneira, percebe-se a necessidade de estudos de avaliação econômica da ECMO em nosso meio a fim de sustentar a sua inclusão como um procedimento pago pelo SUS.

A partir do presente relato, sugerimos o tratamento de resgate precoce com ECMO venovenosa de pacientes pós-pneumonectomia que desenvolvem SARA com hipoxemia e acidose respiratória refratárias à VM, desde que haja experiência suficiente da equipe médica na condução dessa terapia, que ainda é complexa e tem alto custo.
